# A Novel Microfluidic Platform for Personalized Anticancer Drug Screening Through Image Analysis

**DOI:** 10.3390/mi15121521

**Published:** 2024-12-21

**Authors:** Maria Veronica Lipreri, Marilina Tamara Totaro, Julia Alicia Boos, Maria Sofia Basile, Nicola Baldini, Sofia Avnet

**Affiliations:** 1 Biomedical Science, Technologies, and Nanobiotecnology Lab, IRCCS Istituto Ortopedico Rizzoli, 40136 Bologna, Italy; mariaveronica.lipreri@ior.it (M.V.L.);; 2 Department of Biomedical and Neuromotor Sciences, University of Bologna, 40138 Bologna, Italy; marilina.totaro2@unibo.it; 3 Bioengineering Laboratory, Department of Biosystems Science and Engineering, ETH Zürich, Klingelbergstrasse 48, 4056 Basel, Switzerland; julia.boos@bsse.ethz.ch

**Keywords:** microfluidics, personalized medicine, osteosarcoma, chondrosarcoma, drug screening

## Abstract

The advancement of personalized treatments in oncology has garnered increasing attention, particularly for rare and aggressive cancer with low survival rates like the bone tumors osteosarcoma and chondrosarcoma. This study introduces a novel PDMS–agarose microfluidic device tailored for generating patient-derived tumor spheroids and serving as a reliable tool for personalized drug screening. Using this platform in tandem with a custom imaging index, we evaluated the impact of the anticancer agent doxorubicin on spheroids from both tumor types. The device produces 20 spheroids, each around 300 µm in diameter, within a 24 h timeframe, facilitating assessments of characteristics and reproducibility. Following spheroid generation, we measured patient-derived spheroid diameters in bright-field images, calcein AM-positive areas/volume, and the binary fraction area, a metric analyzing fluorescence intensity. By employing a specially developed equation that combines viability signal extension and intensity, we observed a substantial decrease in spheroid viability of around 75% for both sarcomas at the highest dosage (10 µM). Osteosarcoma spheroids exhibited greater sensitivity to doxorubicin than chondrosarcoma spheroids within 48 h. This approach provides a reliable in vitro model for aggressive sarcomas, representing a personalized approach for drug screening that could lead to more effective cancer treatments tailored to individual patients, despite some implementation challenges.

## 1. Introduction

Personalized medicine aims to revolutionize healthcare by offering treatments tailored to individual patients based on their specific characteristics [1]. In contrast to conventional practices that offer standardized care assuming similarities among “average” patients, personalized medicine customizes treatment to the specific need of each patient. The premise is that this individualized approach can be more effective than a one-size-fits-all method, which may work for some patients, but not for others [2].

Oncogenesis is a multifaceted process influenced by a range of interconnected factors. As cancer advances, genomic alterations result in distinct profiles among patients. Additionally, spatial heterogeneity within tumors, encompassing diverse cancer cell phenotypes, complicates the dynamics of cancer. These variations mirror the evolution of cancer and the impact of therapeutic intervention. Understanding and replicating these crucial aspects is critical for developing models that accurately reflect tumor response and tackle treatment challenges [3,4,5], particularly for aggressive and rare cancer types with poor survival rates, such as musculoskeletal sarcomas.

Personalized medicine uses 3D in vitro and in vivo models derived from patients to predict drug sensitivity for customized therapy [6]. The intricate and adaptable nature of the human tumor microenvironment is not faithfully replicated in 2D preclinical tumor cell lines, making them unreliable for predicting clinical responses. While progress has been made with humanized mouse models, biological disparities between mice and humans prevent a truly authentic representation of human tumors [7]. Patient-derived spheroids spontaneously self-assemble from cells isolated from dissociated native tumor tissue into 3D structures that reflect native hierarchy and biology while retaining patient-specific traits [8]. These spheroids exhibit chemical gradients (oxygen and pH), form a necrotic core, facilitate cell–-cell interactions, and stimulate the secretion of endogenous extracellular matrix (ECM) to mimic the local tumor microenvironment [9]. This makes them more predictive of drug response than 2D cultures [10,11,12]. Recent studies have shown 89% accuracy for patient-derived spheroids of ovarian cancer in predicting responses to first-line therapies [9]. Unlike patient-derived xenografts (PDXs), another preclinical model suggested for personalized strategies, spheroids provide rapid results within days or weeks. They are also scaffold-free, avoiding complexities of cell harvesting from 3D gel models that can affect downstream analyses or introduce artifacts. In summary, these stable, scalable, cost-effective tumor-derived spheroids are useful for compound screening and translational studies with the potential to improve treatment selection, evaluate alternative therapies, minimize treatment delays, reduce suffering, and improve patient outcomes [13].

Various techniques can be used to generate spheroids, but few of them are highly reproducible and manageable with enhanced standardization. Among these, microfluidic devices are highly promising [14,15,16,17,18,19,20]. Microfluidic devices are microscale systems that enable output detection and cell culture at physiologically relevant scales within the same platform. They minimize reagent use, waste, and processing time. These features make them promising platforms to study drug toxicity, efficacy, and pharmacokinetics [21]. Microfluidic devices have emerged as a contemporary approach for cultivating spheroids, employing diverse techniques such as droplet-based methods [22], in-gel spheroid formation [23], non-adherent surfaces within microfluidic systems [24], and purposeful designs to facilitate cell trapping and aggregation [25,26]. Combining microfluidics and patient-derived spheroids can create accurate predictive models for chemotherapy responses. Recent articles emphasize the significance of the microenvironment in determining tumor growth, aggressiveness, invasion, and drug response [27,28,29]. Various elements can be integrated to mimic the native tumor microenvironment, including specific ECM, inorganic materials (e.g., hydroxyapatite) [30,31,32,33,34,35,36], and gradients of oxygen, pH, and nutrients [37,38,39,40]. This tissue-like microenvironment strongly influences spheroid response to drugs, providing reliable tumor-on-a-chip models [41]. Integrating aspects of the tumor microenvironment makes microfluidics valuable for modeling tumor characteristics and screening assays in accordance with the principles of the three Rs (replacement, reduction, refinement) [42], reducing the reliance on animal models. Moreover, the capability to utilize limited cell numbers addresses challenges in screening based on scarce primary tumor cultures [43].

However, few studies have focused on patient-derived spheroids in microfluidics for personalized medicine. A 2019–2024 PubMed search yielded only 56 results, with 30 articles reporting on patient-derived spheroid models in microfluidic microenvironments (keywords “patient derived” AND “spheroid” AND “microfluidic*” AND “cancer” OR “tumor”), with none focusing on aggressive musculoskeletal cancers like osteosarcoma (OS) or chondrosarcoma (CS) [10,44]. Most focused on breast [45,46,47,48], gastric [49,50], and pancreatic cancer [20,51,52], including spheroids in exogenous ECM to study tumor aggressiveness or angiogenesis. As an example, Jihoon Ko developed a microfluidic gastric cancer model to study patient-derived tumor spheroid-induced angiogenesis [50]. In other cases, researchers studied novel drug effects on patient spheroids, comparing them to PDX models, which are considered standard [53], thereby validating spheroid efficacy for personalized therapy.

Here, we designed a microfluidic device to obtain OS and CS patient-derived spheroids, enabling personalized drug screens. A micro-patterned non-adherent agarose layer mimicked soft peri-tumoral tissues. Rapid spheroid formation and drug testing enabled a quick assessment of potential chemotherapy outcomes. We validated the performance of the developed model using image analysis.

## 2. Materials and Methods

Consumables, instruments, chemicals, and drugs used in this study are reported in Table 1.

### 2.1. Design and Development of the Microfluidic Device

The microfluidic device comprises two layers of polydimethylsiloxane (PDMS): a bottom layer housing two spheroid culture chambers bonded to a glass slide, and an upper layer with the culture medium reservoir. PDMS was selected for its advantageous properties, including elasticity, optical transparency, biocompatibility, low autofluorescence, thermal and chemical stability, patterning technologies like replica molding, and soft lithography [54]. Combined with rapid prototyping, this enables swift, cost-effective development of microfluidic structures [55]. The bottom layer was fabricated from a 3D printed master mold (ceramic-like Perform material) by using standard soft lithography. The mold design was created in Autodesk^®^ Inventor Professional 2024 software and then it was 3D-printed by stereolithography. Briefly, PDMS and a curing agent were mixed at a 10:1 *w*/*w* ratio, degassed, and poured on the master mold. After 3 h curing at 60 °C, the PDMS layer was detached from the 3D master mold and bonded to a glass slide by plasma bonding using a plasma bonding pen. The bottom layer contains the culture chamber and microfluidic channels connecting the culture chamber to medium reservoirs. The upper layer was obtained by pouring the PDMS solution onto a flat surface. After 3 h curing at 60°, the PDMS layer was peeled off from the flat surface, cut with the same dimension of the bottom layer, and punched with a 4 mm biopsy punch to obtain the culture medium reservoirs. Prior to bonding the two PDMS layers, an agarose solution (2% *w*/*w* in PBS, low-melting-point agarose) was loaded into the culture chamber of the bottom layer to obtain a micropatterned surface thanks to another 3D-printed master mold to favor cell aggregation and spheroid formation. We chose agarose as the hydrogel to use in our system because it facilitates the diffusion of nutrients, oxygen, and metabolites during cell culture [56] and enables easy inverse gelation modeling to create conical microwells. When introduced above 50 °C, agarose can be molded and cooled, retaining its microstructured pattern, which is essential for spheroid cell culture incubation. The agarose mold was designed with cone-shaped microwells (10 wells/culture chamber) in Autodesk Inventor, 3D-printed via stereolithography (Protolabs), and placed on the bottom layer with wells facing downward. Liquid agarose (2% *w*/*w* in PBS) was dispensed through a hole in the mold, cured at −20 °C for 5 min, and the mold was removed. Dimensions of 2 mm deep and width per cone were verified by stereomicroscopy (SM Z18, 1× and 2× objectives). The agarose structure was then washed with antibiotic solution (20 U/mL penicillin, 100 mg/mL streptomycin solution in 5% *v*/*v* in PBS). The PDMS layers were bonded using uncured PDMS as glue, as previously described [19]. The glue (10:1 PDMS–curing agent *w*/*w*) was spin-coated on a 50 × 70 mm glass slide at 2000 rpm for 200 s. Both PDMS layers were brought into contact with the uncured glue and each other. The assembled chip was left in a humid environment at 37 °C 48 h for glue to cross-link. Pre-use sterilization occurred under UV light for 1 h.

#### Fluid Dynamics: Qualitative Evaluation

To qualitatively evaluate the fluid dynamics within the microfluidic device, an erythrosine B solution (0.04 g/100 mL of distilled water) was employed. A 200 µL volume was manually introduced through the inlet, and flow characteristics were monitored under static and dynamic conditions to detect leaks and ensure adequate channel perfusion. For the dynamic setup, the device was positioned on a bidirectional agitator (Organoflow^®^) to promote passive liquid flow at a 3° incline with a 10 min interval to optimize performance.

### 2.2. Cell Culture

OS and CS cells were previously isolated from patient biopsies by mechanical and enzymatic digestions and by culture passages and characterized [57]. Institutional ethics approval and patient informed consent were obtained before sample collection (Prot. 0033626, 9 November 2011). Cells were cultured in Eagle’s minimum essential medium supplemented with 10% fetal bovine serum (Sigma), 20 U/mL penicillin, and 100 mg/mL streptomycin. Cells were maintained at 37 °C in a 5% CO_2_ humidified atmosphere and used at passage 4. We then obtained the 3D cell culture in the microfluidic device. For this, OS and CS cells were cultured on a standard 2D plastic support until reaching 90% confluence. Cells were then trypsinized and assessed for viability (erythrosine B staining), and 200 µL of cell suspensions were separately loaded into the microfluidic devices through the inlet (5.000 cell/well). Within 4 h after seeding, cells had settled at the bottom of the agarose wells. Devices were maintained at 37 °C in a 5% CO_2_ humidified atmosphere, and after 24 h were placed on the OrganoFlow^®^ for passive perfusion (3 °C inclination with 10 min interval) for 72 h to allow spheroid formation and growth. To proceed with the drug screening, OS and CS spheroids (5.000 cells/well) were then obtained as previously described and exposed to increasing doses of DXR (0–5–10 µM), with 20 spheroids per dosage condition. At 24 h after seeding, the culture medium was replaced with 200 µL of fresh medium containing DXR and incubated for an additional 48 h.

### 2.3. Quantification of Spheroid Size Through Transmitted Light Images

To monitor the growth kinetics of OS and CS spheroids over time, daily transmitted light microscopy images of spheroids were captured (objective 10×). Spheroid diameter measurements were obtained with ImageJ software (version 1.8.0, U.S. National Institute of Health, Bethesda, MD, USA) using maximum and minimum diameters and the calculated medium diameters at different time points after seeding and in different conditions, with 20 spheroids (5.000 cells/well) for each condition.

### 2.4. High-Throughput Fluorescent Imaging

To evaluate OS and CS spheroid responsiveness to DXR treatment, spheroids were stained with fluorescent dyes 48 h post-DXR and imaged using an ImageXpress PICO high-throughput automatic imaging system (objective 4×). This system enables simultaneous imaging of the entire spheroid chamber (n = 10 spheroids) in under 10 min. Spheroids were stained with a solution of calcein AM (live cells, 5 µg/mL) and Hoechst 33342 (0.125 μg/mL, nuclear staining, live and dead cells). Spheroids (n = 10 per condition) were incubated with 200 µL of staining solution for 40 min at 37 °C, protected from light.

#### Measurement of Calcein AM Signal in Spheroid Cultures

To measure spheroid viability, images captured 48 h post-DXR treatment (0–5–10 µM) were analyzed. Spheroids were rinsed with PBS and imaged in the ImageXpress PICO system (green channel for calcein AM; blue channel for Hoechst 33342). Exposure of 100 ms was set for calcein AM and 30 ms for Hoechst 33342. Quantification was performed on 10 spheroids for each condition using different methods: (a) ImageJ analysis considering only the calcein-positive area, regardless of fluorescent intensity. The green-fluorescent region was assumed to correspond to live spheroid area. Maximum (D_max_) and minimum (D_min_) diameters of the green-fluorescent portion were manually measured; and (b) analysis by NIS Elements AR 5.40.01 (Nikon) considering both area and fluorescence intensity levels, assuming high intensity denoted regions with more live cells. A region of interest (ROI) covering the green, fluorescent area was automatically recognized. Within this, a distinct area with green fluorescence intensity above 100 relative fluorescence units (RFUs) (40% of maximum) was recognized as the binary area. From the binary area, we then derived the binary area fraction (BAF) that corresponds to the percentage of the detected binary area in respect to the ROI. Due to discrepancies stemming from inherent differences between the two methods and cell lines, a combined parameter called the indirect and mediated vitality index of the spheroid (IMVIS index) was developed and individually calculated for each spheroid (n = 10):

D_mean_ = (D_min_ + D_max_/2)

V = 4/3 **×**π **×** (D_mean_/2)^3^

IMVIS = V **×** BAF

where D_min_ and D_max_ are the minimum and maximum diameter of each spheroid and V is the volume assuming that each is a sphere with diameter corresponding to the mean diameter (D_mean_).

### 2.5. Statistical Analysis

For statistical analysis, GraphPad Prism (version 7.05, GraphPad Software, Boston, MA, USA) was used. Data did not pass the normality test (D’Agostino–Pearson omnibus normality test), and thus differences between groups were analyzed by two-tailed unpaired nonparametric Mann–Whitney U test. All values are expressed as means ± standard error of the mean (SEM). *p* < 0.05 was considered statistically significant.

## 3. Results and Discussion

### 3.1. Fabrication of the Microfluidic Device

Recently, polymethyl methacrylate (PMMA) and polyethylene glycol (PEG) have become popular in microfluidics due to their reduced interactions with hydrophobic drugs, making them suitable for specific biomedical applications [58]. However, PDMS remains the most used material because of its flexibility and ease of fabrication.

**Figure 1:**
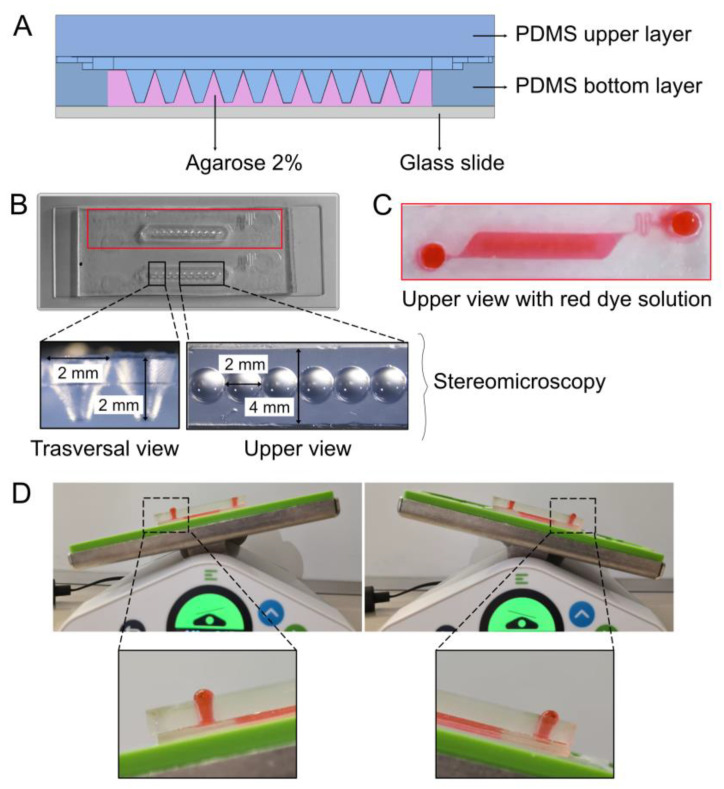
Development and characterization of the spheroid-containing microfluidic platform. (**A**) Schematic of the microfluidic device, comprising a PDMS bottom layer bonded to a glass slide, a microstructured agarose compartment with cone-shaped wells on the surface, and an upper PDMS layer. (**B**) Image of the assembled microfluidic device. On the bottom, enlarged images acquired by stereomicroscopy of the cone-shaped wells (transversal view, 2×, Upper view 1×). (**C**) A red dye loaded in the microfluidic device proves absence of leakages at the macroscopic level. (**D**) Images of the microfluidic device on the OrganoFlow^®^ showcase the correct fluid flow and the absence of leakages during the tilting process at a macroscopic level.

We designed a PDMS–agarose microfluidic device for spheroid formation and drug screening using patient-derived cancer spheroids, particularly for rare and highly malignant musculoskeletal cancers with poor survival, in support of a personalized medicine approach. The device’s dimensions are compatible with standard imaging slides for live spheroid imaging and analysis. This device consists of two PDMS layers bonded onto a glass slide (Figure 1A) to ensure compatibility with the microscope stage. The bottom layer features two parallel spheroid culture chambers, each containing ten agarose cone-shaped microwells (Figure 1B). This parallel design allows for assay replication. Although agarose is commonly utilized for spheroids [59,60,61,62,63,64,65], its application in microfluidics to generate/grow tumor spheroids is limited. The microwells were specifically designed with a width and depth of 2 mm, a −20 degree taper angle, and a 0.5 curvature (Figure 1B). These dimensions were validated by imaging and ImageJ software analysis. Each chamber is connected to the inlet and the outlet reservoir via a meandering microfluidic structure, which also provides fluidic resistance to reduce shear stress during tilt-induced flow [19] (Figure 1B,C). When creating the microwells, we considered the significant influence of well geometry on cell aggregate formation and manipulation [66]. A low diameter-to-depth ratio and shallow depth facilitate easy spheroid harvesting, whereas a higher ratio is more suitable for long-term culture. Cubical or cylindrical shapes enhance oxygen and medium delivery to the cell aggregates. The microwell design and the 4 mm array width were chosen to ensure that cells seeded are effectively captured within the microwells, preventing them from dispersing on the agarose surface. This approach minimizes cell loss and maintains the desired seeding density. Dye loading tests showed no leaks from the PDMS glue bonding (Figure 1C) and proper fluid flow through microfluidic channels and culture chambers during tilting, as evidenced by dyed fluid bubbles in reservoirs (Figure 1D).

In conclusion, we developed a device that includes microfluidic components micropatterned from PDMS, with agarose providing a low-adhesion surface that supports scaffold-free cell aggregation.

### 3.2. Formation of Patient-Derived Spheroids in the Microfluidic Device

After device fabrication, we used it for the drug screening of patient-derived spheroids of OS and CS. Currently, there is a lack of reports on obtaining spheroids from patient-derived musculoskeletal cancers, likely due to the rarity of these tumors. OS is the most prevalent primary malignant bone tumor, while CS ranks as the second-most common, distinguished by its abundant production of cartilage-resembling ECM. Survival rates for metastatic patients are extremely low, with less than 20% for OS [67] and under 40% for CS [68]. The complexity of these cancers has resulted in a lack of precise preclinical models for predictive drug screening, hindering therapeutic advancements over the last decade. Consequently, 3D models that more accurately mimic tumors are critically required.

**Figure 2:**
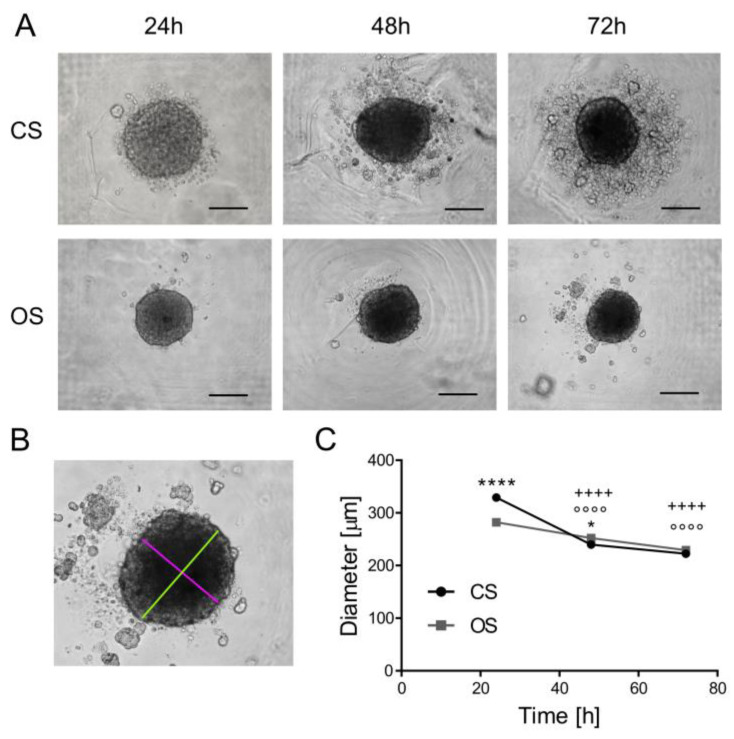
Formation, growth, and characterization of OS and CS spheroids. (**A**) Representative images of spheroids (transmitted light microscopy) at 24–48–72 h post seeding in the microfluidic device (magnification 10×, scale bar 200 μm). (**B**) Representative image of spheroid size measurement, where the maximum diameter (green line) and minimum diameter (red line) were manually traced and calculated using ImageJ software. (**C**) Graph of the diameters of the spheroids, obtained by manual quantification (ImageJ software). Means ± SEM (Mann–Whitney U test, * *p* < 0.5, **** *p* < 0.0001, OS vs. CS at the respective time points, and ^++++^ *p* < 0.0001 for CS vs. 24 h, °°°° *p* < 0.0001 for OS vs. 24 h).

Suspensions of patient-derived OS and CS cells were separately introduced into the device. Due to the cell-repellent properties of agarose, cells aggregated in the cone-shaped wells, forming compact, spherical spheroids within 24 h (Figure 2A). OS and CS spheroids remained visible throughout the three-day culture period. At 48 h, some CS cells detached from spheroids, leading to small aggregates in the agarose wells—a phenomenon absent in the OS spheroids. The diameter of spheroids was quantified by ImageJ, measuring and averaging the minimum and maximum diameters (Figure 2B). For both types of spheroids, there was a decreasing trend in diameter over time. The progressive reduction in diameter was likely due to the secretion of ECM, enhancing compactness. The literature indicates that CS produces significant ECM deposition, abundant in glycosaminoglycans and type II collagen [69,70]. In contrast, OS ECM production is influenced by the absence of mesenchymal components [10]. Hence, the higher inherent secretion of ECM may lead to increased CS spheroid compaction. The presence of ECM in patient-derived spheroids is crucial for drug screening assessments as it serves as a barrier to drug penetration, closely resembling the in vivo setting where tumors are enveloped by intricate extracellular surroundings. This attribute enhances the predictive precision of drug responses, providing a better comprehension of how therapies will perform under actual patient conditions.

Lastly, CS spheroids exhibited significantly larger diameters of 327 μm compared to 285 μm for OS (Figure 2C, *p* < 0.0001 at 24 h). However, spheroid sizes tended to converge over time (Figure 2C).

### 3.3. Drug Screening: Spheroid Diameter on Bright-Field Imaging

**Figure 3:**
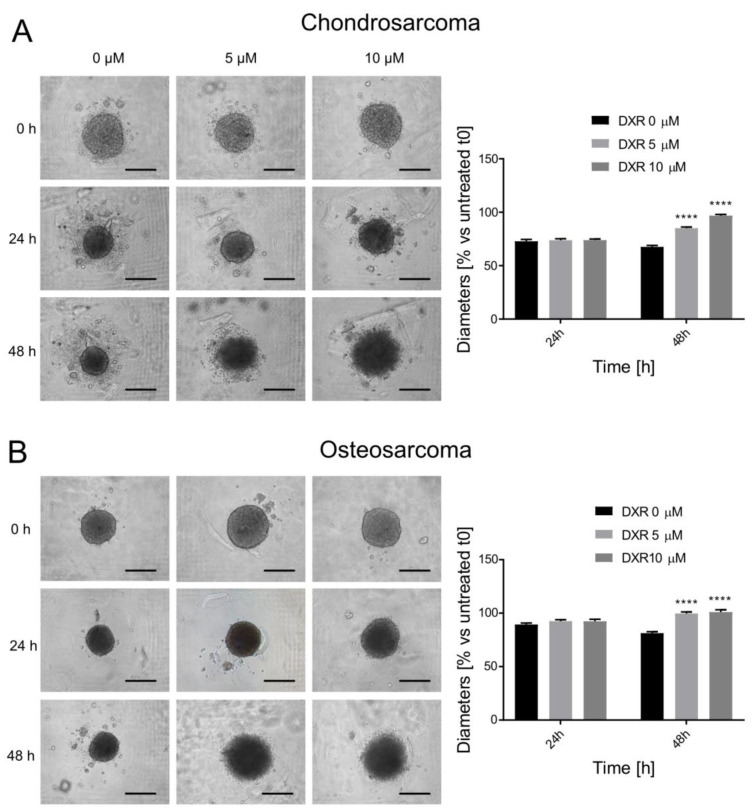
Drug screening assay based on transmitted light images of treated OS (**A**) and CS (**B**) spheroids. On the left, representative images of spheroids before DXR treatment (0 h) and after treatment (24 h and 48 h) at different doses (0–5–10 μM). Magnification 10×, scale bar 200 μm. On the right, graphs of the percentage of DXR-treated spheroid size in respect to untreated spheroids (treated vs. untreated, **** *p* < 0.0001).

After demonstrating the formation of spheroids, screening assays were conducted using the standard chemotherapeutic drug DXR. To facilitate patient-specific drug selection, these assays must rapidly assess chemotherapy responses within a short timeframe, compatible with hospital laboratory settings. Consequently, we identified imaging as a method to assess the effects of chemotherapy on spheroids. The drug screening experiments were conducted by dispensing increasing doses of DXR into the microfluidic device 24 h after cell seeding once the spheroids had developed. Spheroid dimensions were imaged and analyzed at 24 and 48 h post-DXR administration to assess the drug’s effects. At 24 h, the spherical appearance of both cell lines remained largely unaffected by DXR, irrespective of the dose administered, possibly due to insufficient time for duplication or limited drug penetration within the 3D spheroid structure (Figure 3). Minimal cell detachment was observed solely in CS spheroids, aligning with the quantified diameter measurements. By 48 h post-DXR treatment, a decrease in compactness and less defined boundaries were observable in both spheroid types (Figure 3). This visually looser structure translated quantitatively into significant diameter increases for both cell lines across all DXR doses (*p* < 0.0001). Therefore, DXR may reduce spheroid compactness, resulting in an enlargement of cellular aggregates. Nevertheless, accurately distinguishing between live and dead cells within these aggregates poses a challenge, and bright-field diameters may not consistently evaluate the effects of chemotherapy.

### 3.4. Drug Screening: Calcein AM Staining and Live Spheroid Diameters

**Figure 4:**
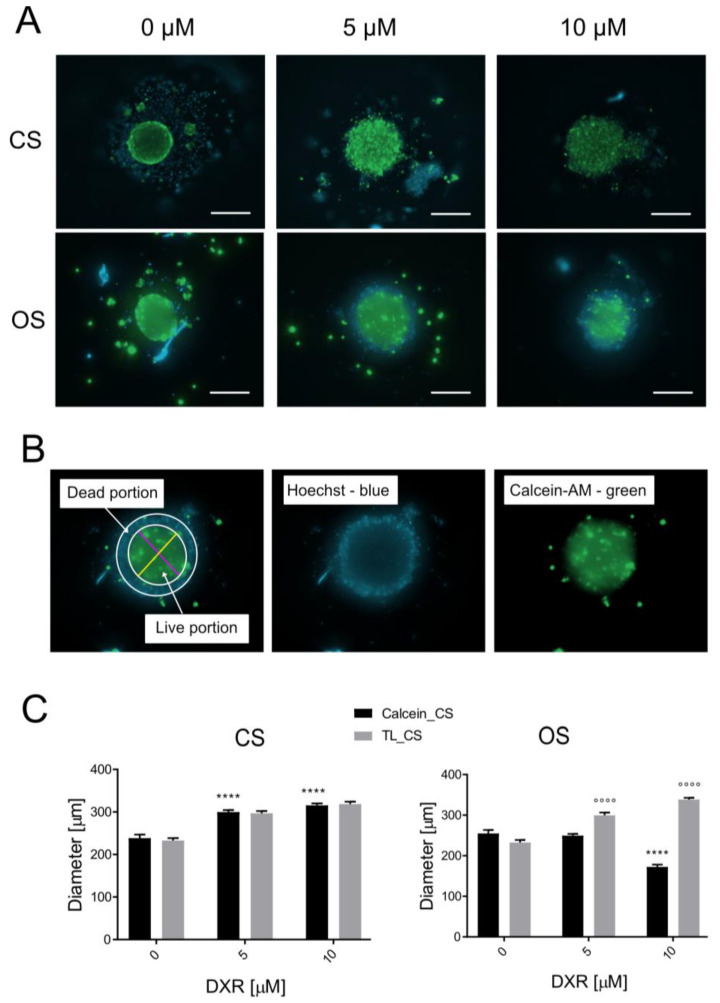
Drug screening assay based on quantification of the fluorescent images of OS and CS spheroids treated for 48 h with DXR (0–5–10 Μm) and positive for calcein AM staining. Hoechst 33342/calcein AM staining distinguished dead cells (Hoechst+ only, blue) from live cells (Hoechst 33342+ and calcein AM+, green). (**A**) Representative images of treated CS and OS spheroids. Images were acquired with ImageXpress PICO machine (magnification 4×, scale bar 200 μm); (**B**) a representative image of spheroid size measurement: maximum diameter (purple line) and minimum diameter (yellow line) manually traced and processed with ImageJ software; (**C**) graph of the average diameter obtained on the calcein AM-positive portion of the fluorescence images (black bars) and on transmitted light images (gray bars) (treated vs. untreated, **** *p* < 0.0001, and diameters measured on bright-field vs. calcein AM signals at the respective DXR dosage, °°°° *p* < 0.0001).

The effect of DXR on spheroid size was further assessed by measuring the diameter of the calcein AM-positive region, representing the viable cell aggregate (Figure 4A). Diameters were calculated by averaging the maximum and minimum measurements (Figure 4B). In the case of CS spheroids, green fluorescence marked the entire spheroid, indicating viability throughout, albeit with a visually less intense and heterogeneous staining compared to controls. Treated CS spheroids appeared less densely packed. In DXR-treated OS spheroids, green fluorescence was predominantly concentrated in the center, with the outer layer of cells staining blue, indicative of cell death. When quantifying diameter, this outer layer of dead cells was excluded. Notably, the diameter of CS spheroids significantly increased in treated spheroids compared to controls, with no variance between doses (Figure 4C). Since calcein stained the whole spheroid, the diameters derived from fluorescent images corresponded to those from bright-field images. Adversely, for OS spheroids, a 5 μM DXR concentration showed no impact, whereas a 10 μM DXR concentration significantly reduced spheroid diameter (Figure 4C). Furthermore, the comparison between bright-field and calcein-based diameters revealed a significance difference in measurement methods for both doses (*p* < 0.0001).

In summary, we observed contrasting behaviors: CS spheroids appeared entirely viable, while OS spheroids exhibited central viability surrounded by areas of cell death. This discrepancy could potentially stem from varying levels of endogenous ECM secretion. The ECM produced by CS might impede drug penetration, leading to resistance [10], whereas the peripheral cell death in OS spheroids could facilitate better diffusion without encountering matrix barriers. This is supported by the significant difference in diameter measurements of treated spheroids, which was observed only in the case of OS. However, it is worth noting that the fluorescence intensity of DXR-treated spheroids varied across different areas of the spheroids, potentially reflecting differences in live cell distribution or levels of viability.

### 3.5. Drug Screening: Calcein AM Staining and Live Spheroid Diameters and Fluorescence Intensity Parameters

**Figure 5:**
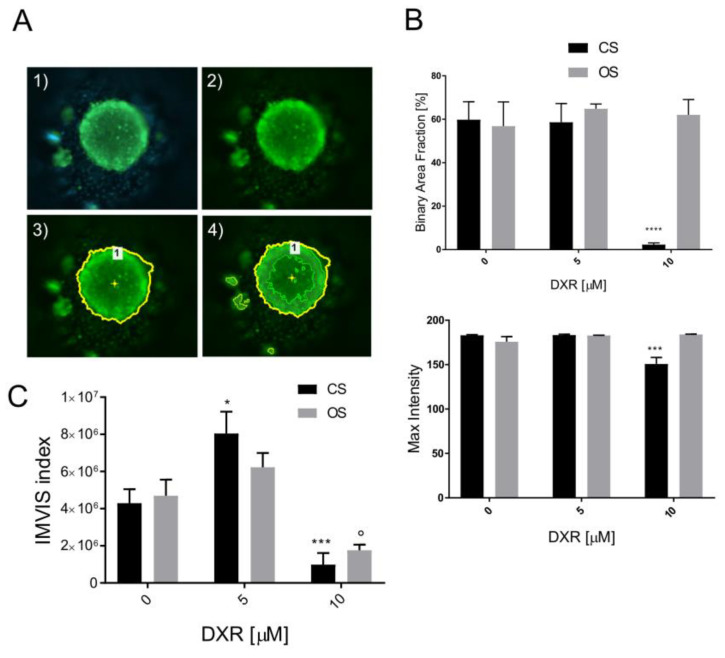
Drug screening assay based on quantification of the BAF of highest intensity range for calcein AM staining of OS and CS spheroids, treated for 48 h with DXR. (**A**) Schematic of method to calculate the BAF and maximum intensity: (1). load fluorescence image for NIS Elements AR 5.40.01 software analysis; (2). select green fluorescence channel: (3). select ROI, corresponding to the green-stained spheroid area; (4). define area within ROI where green channel fluorescence exceeds selected threshold. (**B**) Graphs showing BAF and the maximum intensity for DXR treated vs. untreated spheroids (*** *p* < 0.001, **** *p* < 0.0001). (**C**) Graph of IMVIS of DXR-treated spheroids, calculated by multiplying calcein AM-positive assumed volume and BFA (treated vs. untreated, * *p* < 0.05, *** *p* < 0.001, ° *p* < 0.05).

Therefore, we also examined the calcein AM fluorescence intensity of spheroids treated with increasing doses of DXR for 48 h. We set up identical excitation/emission parameters for the examined spheroids at different DXR doses and for the two sarcoma isotypes and established a threshold for the green (live) signal. Specifically, we calculated the BAF ratio by determining the spheroid area with the highest intensity values, assumed to contain the most live cells, over total fluorescent area (Figure 5A). In the case of OS spheroids, the BAF remained constant regardless of the DXR dose applied (Figure 5B). However, for CS spheroids, the BAF drastically decreased at the highest 10 μM dosage (Figure 5B). Similar trends were observed for the maximum detected fluorescence intensity, with a significant effect only in CS spheroids at the 10 μM DXR dose (Figure 5B). These findings suggest that some green-marked CS cells may not be entirely viable, possibly entrapped within the dense ECM.

Given the divergent responses to DXR treatment between the two types of sarcoma, depending on their specific characteristics, we propose the IMVIS. This integrates two parameters—the calculated calcein AM-positive spheroid volume and the BAF—to assess the effects of chemotherapeutics. This innovative method combines the calcein-positive spheroid volume with the BAF value, encompassing both fluorescence positive signals and intensity. The IMVIS significantly increased only for CS at the 5 μM dose compared to controls, likely reflecting reduced compactness and cell–cell contact. Conversely, at the higher 10 μM dose, a decrease in IMVIS was observed for both OS and CS (Figure 5C). As a result, the use of the microfluidic device for generating patient-derived spheroids and the IMVIS allowed for the rapid assessment of chemotherapy effects without the need for additional time-consuming metabolic assays relying on spheroid lysates or supernatants. As a result of the use of the IMVIS, we were able to assume that CS spheroids were also sensitive to DXR, even in the presence of endogenous ECM, although cell death may have been less rapid compared to OS cells, possibly due to slower permeation of DXR through the spheroids.

In this study, we utilized previously isolated patient-derived 2D cancer cell cultures selected based on their proliferation and adhesion capabilities, as well as culture passages. However, to comprehensively validate and enhance the clinical applicability of this system, future research should include larger samples, comparison with standard viability methods (such as alamar blue or MTT), and integration with a well-defined rapid system for isolating tumor cells or mixed cell populations sourced from tumor biopsies within hospital laboratories. This integration would aim to increase the real-world applicability of the device, making it more scalable and efficient. For instance, combining the device with a sorting system, such as dielectrophoresis and flow cytometry [71,72], could directly extract tumor cells from biopsies. Recent advancements have enabled the implementation of these techniques at the microscale through microfluidic devices, a field known as multiphysics microfluidics, characterized by high precision, sensitivity, real-time tunability, and multi-target sorting capabilities [73]. Thus, microfluidics holds promise in the realm of personalized medicine, serving not only for drug screening but also for tumor detection [74,75]. Advanced devices now incorporate optical biosensors, like surface plasmon resonance, to enhance detection accuracy and automation [76,77].

Conversely, a progressive step forward may be represented by creating spheroids that closely mimic the in vivo tumor heterogeneity and multicellularity of the patient, starting with a pure mixed cell population isolated directly from the tumor [7,9]. This type of spheroid is named a patient-derived organoid (PDO) [78]. By retaining both tumor cells and microenvironmental cells in the in vitro tumor model, these models replicate the complexities of real tumor tissue, as extensively discussed by previous authors [79,80]. In both cases, it will be crucial to assess the device’s scalability for a broader range of tumor types and regulatory aspects [81,82,83].

## 4. Conclusions

**Figure 6:**
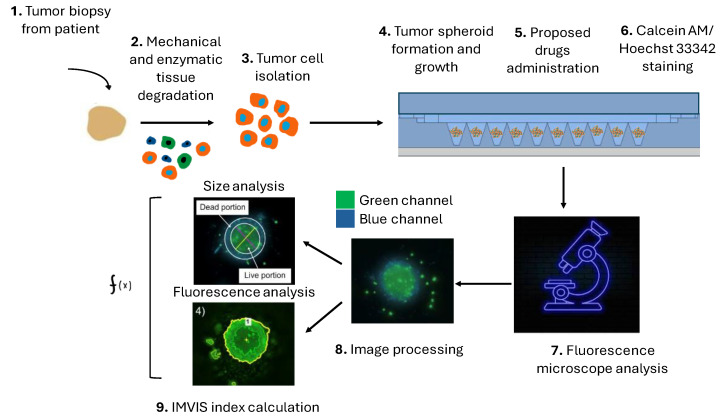
The step-by-step process envisioned for the personalized therapeutic approach, founded on our microfluidic device for generating patient-derived spheroids and their image analysis, progressing from patient’s bedside to tailored outcomes on individualized anticancer therapies. First, a biopsy is taken from the patient (1), it is mechanically and enzymatically degraded (2) and only tumor cells are isolated (3). The tumor cells are then seeded in the platform (4) to obtain spheroids from patient cells and, once the spheroids are formed, a chemotherapeutic drug is administered (5). After the treatment, a live staining is performed (6), and images are subsequently acquired using a fluorescence microscope (7), processed (8), and analyzed with IMVIS index (9) to evaluate the proportion of live and dead cells within the spheroid.

We presented a novel PDMS–agarose microfluidic device that effectively facilitates the rapid formation and growth of tumor spheroids starting from patient-derived tumor cell population for personalized drug screenings. In our model, bright-field spheroid diameter calculations failed to predict DXR cytotoxicity in OS and CS spheroids. As a result, we propose a new spheroid viability index integrated with the device, incorporating: (1) signals exclusively from live cells; (2) volume occupied by live cells; and (3) fluorescence intensity. We validated this index on patient-derived OS and CS spheroids exhibiting varying DXR sensitivity. This method offers a personalized tool tailored to each patient’s tumor for drug screening, following a systematic process from the patient’s bedside to the resultant outcome on potential personalized anticancer treatments (Figure 6). Although implementation challenges exist, we envision integrating this device into treatment planning in order to enable effective, personalized anticancer care.

## Figures and Tables

**Table 1:** Consumables, instruments, chemicals, and drugs used in this study.

Category	Product	Vendor	Country
Materials	Silicone elastomer Sylgard 184	Dow Corning Corp.	Midland, MI, USA
	Curing agent Sylgard 184		
	3D-printed master molds	Protolabs	Feldkirchen, Germany
Instruments	Plasma bonding pen	Elvesys	Paris, France
	Organoflow^®^ agitator	Mimetas	Leiden, The Netherlands
	ImageXpress PICO	Molecular Device	San Jose, CA, USA
	Stereomicroscope SM Z18	Nikon	Tokyo, Japan
	Transmitted light microscope ECLIPSE—TE 2000-S		
Reagents	Low-melting-point agarose	Sigma	St. Louis, MI, US
	Erythrosine B		
	Eagle’s Minimum Essential Medium		
	Fetal bovine serum		
	Hoechst 33342		
	Penicillin–streptomycin solution	Euroclone	Pero, MI, Italy
	Calcein AM	Thermo Fisher Scientific	Waltham, MA, USA
Drug	Doxorubicin (DXR)	Sigma	St. Louis, MI, US

## Data Availability

Data are unavailable due to privacy restrictions.

## Abbreviations

3D	tridimensional
2D	bidimensional
ECM	extracellular matrix
OS	osteosarcoma
CS	chondrosarcoma
PDMS	polydimethylsiloxane
DXR	doxorubicin
D_max_	maximum diameter
D_min_	minimum diameter
D_mean_	mean diameter
ROI	region of interest
RFU	relative fluorescence unit
BAF	binary area fraction
IMVIS index	indirect and mediated vitality index of the spheroid
V	volume
SEM	standard error of the mean
h	hours
PDXs	patient-derived xenografts
PMMA	polymethyl methacrylate
PEG	polyethylene glycol
